# Biologic Pollution Due to *Ambrosia* (Ragweed) Pollen in Urban Environment of Bucharest

**DOI:** 10.3390/ijerph191710613

**Published:** 2022-08-25

**Authors:** Polliana Mihaela Leru, Vlad Florin Anton, Ana Maria Eftimie, Sorin Stefanut

**Affiliations:** 1 Clinical Department 5, Carol Davila University of Medicine and Pharmacy, 050474 Bucharest, Romania; 2 Department of Allergology, Colentina Clinical Hospital, 020125 Bucharest, Romania; 3 Department of Ecology, Taxonomy and Nature Conservation, Institute of Biology Bucharest, Romanian Academy, 060031 Bucharest, Romania

**Keywords:** aeroallergens, air pollution, *Ambrosia* (ragweed) pollen, biologic pollution, urban environment

## Abstract

Ragweed pollen is an important component of biological pollution in the urban environment, responsible for increasing respiratory allergies and significant contribution to the health impact of air pollution in the Bucharest area. The aim of this paper is to present the eight-year ragweed pollen monitoring data from Bucharest, to place them in the context of local air pollution, public health regulations and available data on the health impact of ragweed pollen in the urban environment. Our pollen data were correlated with major air pollutant concentrations and with meteorological factors in a recently published local paper and the clinical data of patients with ragweed-induced respiratory symptoms were collected and published in 2019. The ragweed pollen monitoring data, correlated with field data reported by patients and plant specialists confirm the rapid spread of *Ambrosia* in the Bucharest city area, in addition to some stringent environmental local problems due to air pollution. The number of patients addressed to allergists almost doubled from one year to another, confirming the real alarming health impact of this environmental hazard. Our study confirms the need for more coherent strategies to control ragweed spread, based on application of existing local and international regulations, air pollution control and evaluation of consequences on human health.

## 1. Introduction

The previous decades witnessed an epidemic increase in allergic respiratory diseases, whose manifestations and severity are aggravated by environmental factors and air pollution [1].

Biological air pollution is partly caused by pollen grains originating from allergenic plants, which can preferentially contribute to the sensitization and development of atopic illnesses such as allergic rhinitis and asthma [2]. Consistent research was focused on understanding the mechanisms of interaction between pollen, air pollution, climate change, and how these environmental factors influence the development and severity of allergies, aiming to find and implement counteracting management tools. Recent studies showed that anthropogenic climate change is likely to affect the severity of allergies by altering the chemical, physical and biological composition of the atmosphere and also the timing and intensity of the pollen season [3,4].

*Ambrosia artemisiifolia* (common ragweed) is a rapidly extensive invasive weed in many European countries, representing an important environmental and health hazard [5]. *Ambrosia* is a native plant in North America, first noted in Europe during the second half of the 19th century and was initially imported to Central Europe after the First World War [6]. This invasive weed has accelerated its expansion in Europe, currently, the species is widespread in southern parts of Eastern and Central Europe, with invasion hotspots in the Pannonian plains of Hungary [7], Croatia, Serbia, Italy, Ukraine [8], Russia [9], and France [10,11]. After the 1980s, *Ambrosia* spread rapidly increased and became a serious threat to human health in the infested countries. Hungary, Northern Italy, and Rhone Valley from France are considered the most infested European regions and intense research activities have been dedicated to this topic [12]. The main factors favoring *Ambrosia* spread are considered inappropriate changes in agricultural practices, urbanization, and the international trade of seeds and grains [13].

The increase in the *Ambrosia* atmospheric pollen load and the size of the affected areas have been consistently recorded, according to the data issued from the European pollen count database, which was established in 1988 [14]. Many studies confirmed that *Ambrosia* has become increasingly important from an allergist point of view, covering large areas of Central and Eastern Europe [15]. According to allergists’ medical records, almost 40% of the European population suffers from pollen allergies and the percentage of patients who are sensitized to *Ambrosia* pollen is steadily rising, ranging from about 30% in France and Austria up to 80% in Hungary [5,16]. It is largely known that climate change, urbanization, and its long-distance transport capacity enhance the spread of the weed and increase the allergenicity of its pollen [17].

It was demonstrated that climate change and rapid urbanization induce increasing temperature and CO_2_ concentrations and have great influences on *Ambrosia* pollen production, leading to higher rates of sensitization in exposed populations [18]. The most important impact of *Ambrosia* in Europe is on human health, causing diverse allergic symptoms such as rhinitis, hay fever, asthma, and dermatitis. Furthermore, it infests arable crops causing substantial yield loss [19].

Romania is considered one of the European countries with high *Ambrosia* infestation rates, and this plant is now seen as a serious threat to both human health and the environment, raising significant public concern [20,21].

The aim of this paper is to present relevant data regarding biological pollution due to *Ambrosia* in Bucharest capital city of Romania, based on pollen monitoring data and related research during the last eight years, and to place them in the context of local air pollution. We also aimed to evaluate the implementation of national regulations and available data on *Ambrosia’s* health impact in the urban environment and to outline some unsolved issues regarding air pollution in Romania.

## 2. Material and Method

We used the most relevant literature data regarding *Ambrosia* spread in Romania and illustrated with the map showing the national wide distribution mostly in the plain regions from west, south, and east. The map was created using the Universal Transverse Mercator (UTM) technique, botanical literature, public herbarium data, and the author’s own field observations.

We added a recent map showing distribution of *Ambrosia* in Bucharest, performed based on field observation of the plant specialist from the Institute of Biology.

The atmospheric pollen measurement and monitoring were performed in accordance with the recommendations of the European Aerobiology Society (EAS), using a Hirst-type volumetric pollen trap set up at a height of 19 m on the roof of a four-level building from Colentina Clinical Hospital [22]. Pollen grains were sampled continuously, the sampling airflow rate was 10 L/min, analysis was performed weekly, and daily pollen concentrations were expressed as particles per cubic meter of ambient air (P/m^3^).

In a local recent study on the correlation between pollen, air pollutants, and meteorological factors, the measurement daily data of major air pollutants: particulate matters with diameters of 10 μm and 2.5 μm (PM_10_ and PM_2.5_), nitrogen oxides (NO_x_), carbon monoxide (CO), volatile organic compounds (VOC_s_), ozone (O_3_), sulfur dioxide (SO_2_) were obtained from the Bucharest Air Quality Monitoring Network [23]. The meteorological data: temperature, relative humidity, air pressure, solar radiation, and daily precipitation, were provided by National Meteorology Administration, Filaret Station of Bucharest.

## 3. Results

### 3.1. Data Regarding Ambrosia Spread in Romania

*Ambrosia* was first reported in Romania in 1908, based on herbarium specimen from the southwest city of Orșova [24] from where this invasive weed rapidly spread towards the west, center, and south, presently being a permanent presence in Romanian flora [25,26]. Recent field observations performed in different regions of the country and clinical reports from allergists confirmed the rapid and extensive spread of *Ambrosia* to large plain and hill regions, from all Romanian provinces [27,28].

**Figure 1:**
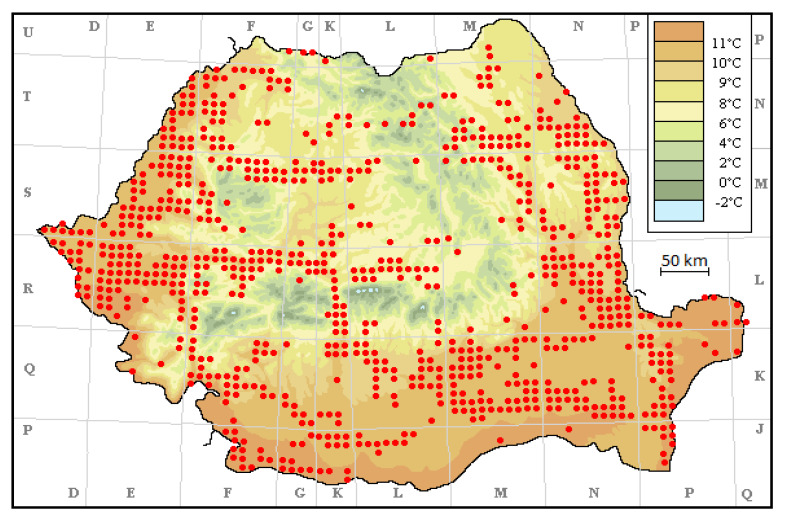
The map of *Ambrosia* spread in Romania between 1908–2012 (Culita Sirbu, 2012, original work, unpublished, reproduced with author’s permission), completed with mean annual temperature by Sorin Stefanut (UTM system, 10 × 10 km).

The distribution of *Ambrosia* in Romania from the time of its first report in 1908 until 2012 is shown in Figure 1.

*Ambrosia artemisiifolia* species are short-day plants, producing very large amounts of pollen during the flowering period in August–September and even small populations can be responsible for an increased pollen exposure. The weed is adapted to low nutrient environments and develops as colonizing crops and ruderal plants in open disturbed habitats, such as roadsides, or riverbanks, and stubble fields, especially in abandoned places around settlements. The major changes in land use in Romania after the fall of the communist regime end of 1989 were favorable to the spread of this weed in urban environments, *Ambrosia* could invade abandoned construction sites, new residential places with deficient infrastructure, and some cultivated land. In combination with disturbance and other environmental stressors which decrease the competition intensity, *Ambrosia* might further spread into more productive environments and its distribution areas could enlarge because of acclimatization in new regions [29].

Intensive research on *Ambrosia* and airborne pollen monitoring previously performed in the western city of Timisoara, demonstrated a high amount of ragweed pollen in that area, exceeding the atmospheric pollen load of all other allergenic plants during late summer–early autumn [20].

Data from the INSPIRED research project (https://oncogen.ro/inspired%20project/, accessed on 21 July 2022) estimated that about 5.35% of the active population of Romania can suffer from ragweed-induced allergies, which is now considered a nationwide issue [30].

In a season pollen monitoring study performed in August 2008, *Ambrosia* pollen was the most important aeroallergen in all of the four urban areas: Timisoara, Cluj-Napoca, Bucharest, and Brasov. In two of these big cities, a comparatively high number of days exceeding the defined threshold value of 20 pollen grains/m^3^ was recorded: 29 for Timişoara and 25 for Bucharest [20]. According to our research on allergenic plants and pollen monitoring, which started in 2013 in Bucharest, we found that *Ambrosia* pollen was a relevant seasonal aeroallergen for the Southeast region of Romania [31].

### 3.2. Distribution of Ambrosia in Bucharest Area

Awareness of *Ambrosia* spread and its health impact on the population of the capital city Bucharest came more than ten years ago from allergists and the public interest in this topic has progressively increased during the last years [32]. Bucharest is the largest town in Romania, situated at 44° latitude and 26° longitude, in the Romanian Plain, at about 60 m altitude above sea level. It has an actual population of about 1,926,000 inhabitants and an area of 228 square km, of which 70% is building area. The local climate is temperate continental, with four seasons, characterized by warm and dry summers. In this region, grass species that pollinate in the spring and summer and weeds that pollinate in the late summer and fall have been reported to be the most allergenic plants.

Bucharest is considered one of the most polluted and crowded European capitals, with many unsolved issues regarding air pollution, in addition to the increasing spread of *Ambrosia* in this area. These weed species have exponentially increased spread in the last years and continue to colonize new territories each year, being notified in all districts from the city center to the periphery.

**Figure 2:**
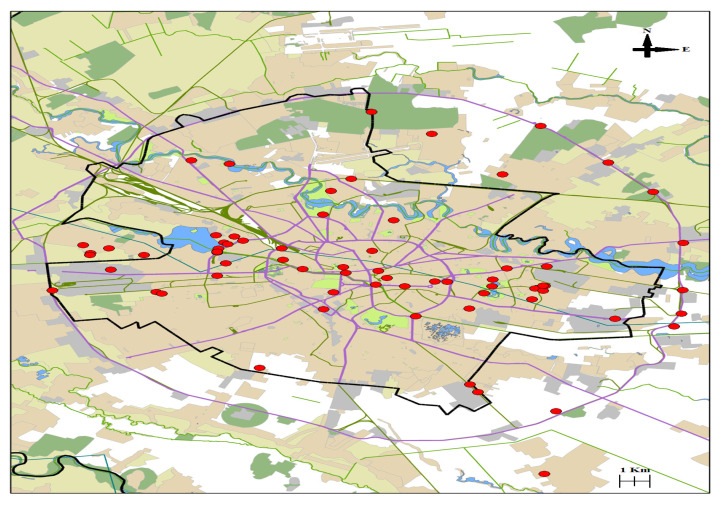
The spread of the weed *Ambrosia artemisiifolia* in Bucharest area (reported by Sorin Stefanut in 2018, original work, unpublished, reproduced with author’s permission), 44° latitude and 26° longitude. The red dots represent notably invaded sites in both public and private properties, the black line delimits the urban area, surrounded by suburban localities and the purple lines indicate the main traffic roads.

An initiative of the Institute of Biology from Bucharest has led to the identification of a large number of sites invaded by *Ambrosia* *artemisiifolia* in all districts of Bucharest, as shown in Figure 2.

### 3.3. Ambrosia Pollen Monitoring Data in Bucharest between 2014–2021

Long-term pollen data for other regions than the western city of Timisoara, were not available until 2014, despite a significant amount of research and scientific literature published by many experts in the field of allergenic plants. There is no national aerobiology network in Romania, and measurements of atmospheric pollen were performed continuously in the west region between 1999 and 2010. Due to our involvement in the COST project dedicated to ragweed, entitled Sustainable Management of *Ambrosia artemisiifolia* in Europe (SMARTER) FA-1203, 2012–2017 (https://www.cost.eu/actions/FA1203/, accessed on 21 July 2022), we initiated the first pollen monitoring laboratory in Bucharest at the Allergology Department from Colentina Clinical Hospital, a large multidisciplinary university hospital situated close to the city center [32]. We started pollen measurement and monitoring on regular basis in Bucharest, which is continuing since 2014, based on our long-term collaboration with the Réseau National de Surveillance Aérobiologique (RNSA) from France and the European Aeroallergen Network. Because of some technical problems, the monitoring was interrupted during August–September 2015. The monthly pollen data were initially transmitted to the RNSA laboratory for validation, and then to the European Aeroallergen Network (EAN) for posting on their website www.polleninfo.org (accessed on 21 July 2022). It was the first time when information about atmospheric pollen from Romania was communicated to European institutions and could be included in further published research papers.

Based on previously published research, we considered that critical threshold values of daily ragweed pollen concentration for provoking symptoms in sensitized patients are below 20 particles/m^3^ air, but this can be much lower in very sensitive patients, as low as 1–5 particles/m^3^ air, which is significantly lower than the threshold of 50 particles/m^3^ air for grasses pollen [33]. We can assume that the *Ambrosia* pollen season in Romania presents similar behavior and duration as in other neighboring European countries, such as Hungary, Serbia, and Croatia, explained by the biogeographical and bioclimatic conditions [34].

**Figure 3:**
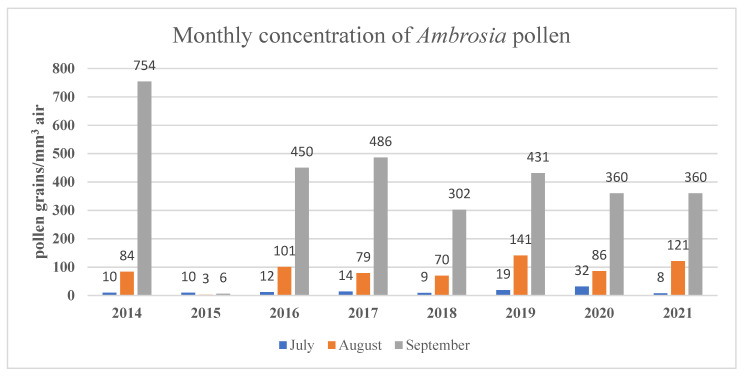
Monthly concentration of *Ambrosia* pollen during July–August–September, 2014–2021.

The results of the monthly *Ambrosia* pollen concentration between May–October are shown in Table 1 and those for the three months ragweed season are shown in Figure 3.

**Figure 4:**
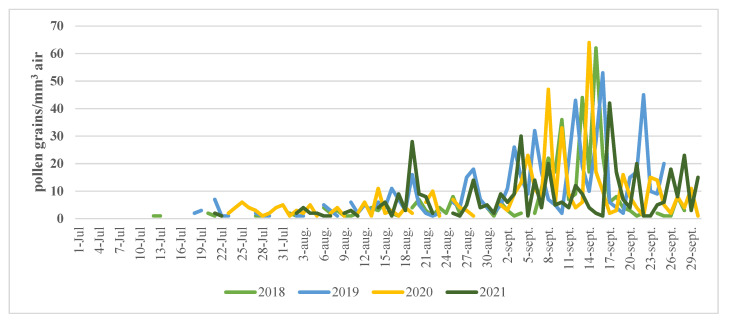
Daily *Ambrosia* pollen concentration during the season between 2018–2021.

We found the highest total monthly amount of 754 *Ambrosia* pollen grains/m^3^ air during September 2014 and the peak of the daily values has reached 231 pollen particles/m^3^ air in early September 2014, which was comparable with the amount of 292 particles/m^3^ air reported in Timisoara in August 2009 [20]. The period with daily pollen concentration above 10 particles/m^3^ of air was from 15th August until the end of September in almost all of the recorded years (Figure 4).

Our air pollen recordings from Bucharest proved that *Ambrosia* is a significant component of the biological pollution in the largest Romanian city from the South region, which is different from previously published studies, claiming that *Ambrosia* is common in Western regions and in rural areas [31]. Note that the year 2014 was the hottest year on Earth since the beginning of the record-keeping in 1880, as mentioned by the Natural Resources Defense Report (NRDC) from the USA in 2015 [35].

The results of the eight-year pollen monitoring in Bucharest provided by our allergy research center have been included in the recently closed European project CAMS_23 of the Copernicus Atmosphere Monitoring Service/ECMWF (The European Centre for Medium-Range Weather Forecasts), coordinated by the European Aeroallergens Network (EAN) and the University of Vienna (www.ean-net.org/en.html (accessed on 21 July 2022). This project aimed to develop and test models that predict the pollen load of four major aeroallergens-birch, grasses, olive, and *Ambrosia* across Europe and its neighboring countries, including 100 pollen monitoring sites.

Since 2017, our laboratory is a partner in the regional project Ragweed Pollen Alarm System (R-PAS), coordinated by the Hungarian Aerobiology Society and the Institute of Public Health from Budapest, running since 2014. This project aims to provide pollen information from countries included or close to the Pannonian biogeographical region, by using a neural network-based ragweed pollen forecast [36].

Our pollen data were correlated with both major air pollutant concentrations and meteorological factors in a recently published local paper, the first one dedicated to this topic in the Bucharest area [23]. Results of this study confirmed a positive correlation between pollen and the most important urban pollutants, such as particulate matter PM_10_ and PM_2.5_, nitrogen oxides, and volatile compounds. For *Ambrosia* pollen, the most notably found correlation was with NO_x_ and PM_10_ concentration.

Interesting data referring to the correlation between pollen concentration and meteorological parameters from the Western region of Romania were presented in another recent local study, showing that *Ambrosia* pollen concentrations was positively associated with high temperature and sunshine hours and inversely associated with relative humidity [37].

### 3.4. Preliminary Health Data of Patients Allergic to Ragweed Pollen

In order to evaluate the health impact and clinical pattern of respiratory allergies induced by *Ambrosia* pollen, we performed a retrospective clinical study and published the first paper on this topic from Bucharest, in 2019 [38]. We collected the clinical data of 760 patients with respiratory allergies who were addressed to our Allergology Department from Colentina Clinical Hospital during 33 months, between January 2017–October 2019, and found that 24.21% (184 patients) were diagnosed with *Ambrosia* induced respiratory allergy. The most frequent clinical pattern was seasonal rhino-conjunctivitis, followed by rhinitis and rhinosinusitis, and 15.21% of the patients were associated with asthma. More than 80% of the cases were moderate or severe clinical forms and 11.95% of the studied group reported a history of atopy. The *Ambrosia* season in this area can last between mid-July to the end of September; therefore, many patients are asking allergist consultation during August- September and have more severe symptoms when the air pollen reaches peak concentration, for about 4–6 weeks. We could not continue and extend the clinical evaluation of allergic patients from Bucharest by involving other allergists, because of the COVID-19 pandemic, therefore these preliminary health data have to be completed. The continuously increasing number of patients with seasonal ragweed-induced respiratory allergies confirms the real alarming health impact of this environmental hazard, in addition to the increased population awareness of this allergenic pollen. The number of patients addressed to our center almost doubled from one year to another. Another recent study comparing clinical data of patients allergic to ragweed pollen from two other big Romanian cities, confirms the increasing number of affected persons from many of the country’s regions [39].

## 4. Discussion

Air pollution is a complex and challenging global problem, with undoubtedly severe consequences for humans and the environment [40]. Its management needs strong and constant determination and implication of political and social decision factors and professional institutions, based on international regulations. An accelerated spread of *Ambrosia* has been observed in Romania since the early 2000s, mostly in the western regions and extending to the south and east [21].

The expansion of *Ambrosia* in Romania, similar to other East European countries, seems to be associated with complex factors mostly due to climate change, but also with major socio-economic transitions, that occurred after the collapse of communism, leading to an increase in areas of disturbed land [41].

The eight-year pollen monitoring data from our center showing high ragweed air pollen concentration during the season, correlated with reported field data confirm the rapid spread of *Ambrosia* in all districts of Bucharest city area, thus anticipating a continuous increasing sensitization rate of the population exposed to ragweed, similar to the situation demonstrated by a 14-year study from Vienna [42]. Based on actual health data, both the number and severity of *Ambrosia*-induced allergies will continue to increase, thus having a great impact on the national health system, increasing direct and indirect costs of medical care. The continuous spread of *Ambrosia* has detrimental environmental consequences, mostly loss of biodiversity by eliminating or outcompeting native species. Since people living in urban areas are 20% more likely to suffer airborne pollen allergies than people from rural areas, administrative authorities from Bucharest have to consider allergist advice, in addition to other environmental specialists, when designing the urban green zones and follow the model from more advanced countries [43].

Taken together, the *Ambrosia* expansion in the urban environment and the stringent local problem due to air pollution, added to higher temperatures and longer hot seasons due to climate change, demonstrate the need for an urgent complex national strategy and efficient control measures. Despite consistent progress recorded in Romania during the last decades in the field of reducing air pollution, there are still many unsolved issues and unmet needs to be considered

## 5. Unsolved Issues and Unmet Needs Regarding Air Pollution in Romania

Referring to biological pollution due to allergenic pollen, Romania is behind other European countries, with still no national aerobiology network and very few pollen monitoring centers, having to accelerate the implementation of local legislation against ragweed and adopt EU strategy [44]. The application of the Law no. 62 adopted by the Romanian Parliament in 2018—On fighting against the invasive common ragweed has still rather modest results, since the species *Ambrosia artemisiifolia*, which control is targeted by these legislative norms is extremely spread nationally, in urban, rural, and borderland areas, as well as in some crop fields [45]. Due to the extent of the common ragweed distribution, checking and identifying the infested areas is a lengthy process. Therefore, the time between its notification and the active measures taken for its control is often enough for the production of this pollen with allergenic potential and then of seeds which help the perpetuation of the species in the next seasons. The current extent of *Ambrosia* infestation has harmful impacts on a range of sectors, especially on human health and agriculture, with economic, social, and environmental effects.

If the control measures against *Ambrosia* are not applied efficiently and coordinated at a national level, this invasive species will continue to spread, triggering serious individual and socio-economic consequences. Based on these observations, it becomes clear that biological pollution can be controlled, and a healthier environment can be provided also by planning urban green spaces, based on vegetation specialists’ advice, aimed to avoid and restrict allergenic species.

Since the early 1990s, Romania recorded real progress in the field of air pollution, by identifying the main problems and implementing the EU acquis into national legislation, reflected by decreasing trend of all major pollutants [46]. Despite these achievements, in 2019 the EU Commission launched an infringement procedure against Romania, directed to some unsolved issues, such as exceedances of PM_10_ and NO_2_ levels, inadequate application of EU Directives, and missing the National Air Pollution Control Program of Romania under Article 6 of EU Directive 2016/2284/EU [47]. The infringement procedure was especially addressed to the capital Bucharest and two other big cities: Brasov (from the Central part) and Iasi (from the Eastern part of the country). The priority actions recommended for Romania refer to the reduction in the chemical air pollutants NO_x_, PM_10,_ and PM_2.5_ and also the improvement of the air quality network, more accurate reporting of air quality data, reduction in coal use, and improvement of EU regulations in this field [48].

Developing the aerobiological monitoring of ragweed and other aeroallergens by implementing new technologies and providing continuous real-time data can be useful for the local and national authorities, clinicians, patients, and the general public in Romania. There is a clear need for more research studies on the correlation between pollen concentration, and air pollution using monitored data collected by Bucharest Air Quality Monitoring Network and meteorological factors.

Information is important both in relation to the forecasting of *Ambrosia* pollen concentrations in the ambient air and also in the design of mitigation strategies because effective strategies vary with the distribution and abundance of allergenic plants. Consistent data from the literature demonstrate that future climate change could result in a large expansion of *Ambrosia*-related allergy problems in Europe, illustrating the importance of cross-countries management planning in order to prevent and control the continuous spread of this dangerouss bio-invader [33].

### Strengths and Limitations

The strengths of this study include the fact that it is based on our almost ten years working in the field of pollen allergies, which is the first and still the only source of pollen data from Bucharest capital city focusing on *Ambrosia*, which is a large interest problem and the most studied allergenic plant. This is the first study in Romania putting together eight-year pollen data from the Bucharest area with recent environmental problems raised by urban pollution and the outcome of new regulations implementation.

The main limitation of our study is that we could not perform an in-depth analysis of climate/meteorological factors with pollen data and major air pollutants, we had only a few local studies to refer to and some preliminary data from Bucharest regarding the health impact of *Ambrosia* pollen.

## 6. Conclusions

We concluded in our study that *Ambrosia* is an important noxious environmental hazard in Romania and its pollen is an important component of biological pollution in the urban environment of Bucharest capital city. It is responsible for increasing respiratory seasonal allergies and has a significant contribution to the health impact of air pollution in this large city area. Due to clear data proving increased of both components of air pollution—biological and chemical in the urban area—there is a need for intensive research and a more coherent strategy to control ragweed expansion and urban pollution, in terms of consistent application of existing local and international regulations, and better evaluation of complex consequences on the environment and human health.

## Figures and Tables

**Table 1:** Total monthly concentration of *Ambrosia* pollen between 2014–2021 (pollen grains/mm^3^ air).

	2014	2015	2016	2017	2018	2019	2020	2021
May		1	2	1			3	
June		1	3			1	2	4
July	10	10	12	14	9	19	32	8
August	84	3	101	79	70	141	86	121
September	754	6	450	486	302	431	360	360
October	4							

## Data Availability

Data are available at Allergology Department, Colentina Clinical Hospital, Bucharest.
